# Anti-Inflammatory Activity of Miodesin™: Modulation of Inflammatory Markers and Epigenetic Evidence

**DOI:** 10.1155/2020/6874260

**Published:** 2020-05-15

**Authors:** Carlos Rocha Oliveira, Rodolfo Paula Vieira

**Affiliations:** ^1^Anhembi Morumbi University, School of Medicine, Avenida Deputado Benedito Matarazzo 6070, Sao Jose dos Campos-SP, Brazil 12230-002; ^2^Federal University of Sao Paulo (UNIFESP), Post-Graduation Program in Sciences of Human Movement and Rehabilitation, Avenida Ana Costa 95, Santos-SP, Brazil 11060-001; ^3^Universidade Brasil, Post-Graduation Program in Bioengineering and in Biomedical Engineering, Rua Carolina Fonseca 235, Sao Paulo-SP, Brazil 08230-030; ^4^Brazilian Institute of Teaching and Research in Pulmonary and Exercise Immunology (IBEPIPE), Rua Pedro Ernesto 240, Sao Jose dos Campos-SP, Brazil 12245-520

## Abstract

**Purpose:**

To investigate the effects of a combined herbal medicine Miodesin™ on the inflammatory response of key cells involved in the acute and chronic inflammatory processes as well as the possible epigenetic involvement.

**Methods:**

After the establishment of the IC_50_ dose, the chondrocyte, keratinocyte, and macrophage cell lines were pretreated for 2 hours with Miodesin™ (200 *μ*g/mL) and stimulated with LPS (1 *μ*g/mL) for 24 hours. The supernatant was used to measure the levels of cytokines (IL-1*β*, IL-6, IL-8, and TNF-*α*) and chemokines (CCL2, CCL3, and CCL5), and the cells were used to extract the mRNA for the transcription factor (NF-*κβ*), inflammatory enzymes (COX-1, COX-2, PLA2, and iNOS), and chemokines (CCL2, CCL3, and CCL5).

**Results:**

Miodesin™ inhibited the release of LPS-induced cytokines (IL-1*β*, IL-6, IL-8, and TNF-*α*; *p* < 0.01) and chemokines (CCL2, CCL3, and CCL5; *p* < 0.01) and the expression of the transcription factor (NF-*κβ*; *p* < 0.01), inflammatory enzymes (COX-1, COX-2, PLA2, iNOS; *p* < 0.01), and chemokines (CCL2, CCL3, and CCL5; *p* < 0.01). In addition, the evaluation of epigenetic mechanism revealed that Miodesin™ did not induce changes in DNA methylation, assuring the genetic safeness of the compound in terms of the inflammatory response.

**Conclusions:**

Miodesin™ presents anti-inflammatory properties, inhibiting hyperactivation of chondrocytes, keratinocytes, and macrophages, involving epigenetics in such effects.

## 1. Introduction

Although the inflammatory process is a natural response to an offending agent aiming to promote healing and repair, an exacerbated and/or unresolved inflammatory process underlies several acute and chronic diseases [1]. The inflammatory process is complex and involves a group of glycoproteins called cytokines, which coordinate, amplify, and regulate the magnitude and duration of inflammatory events [1].

Acute dermatitis and atopic dermatitis are examples of acute and chronic skin diseases, respectively, in which keratinocytes present a key role [2]. In this context, it has already been demonstrated that keratinocytes express different surface alarming receptors against pathogens, being the trigger for cytokines and reactive oxygen species (ROS) and reactive nitrogen species (RNS) release [2, 3]. In addition, lipopolysaccharides (LPS) are among the main players for skin infection, which may be installed during skin acute and chronic inflammatory processes [3]. Beyond keratinocytes, skin macrophages also represent the first line of defense during skin infections, contributing to the inflammatory process, releasing, for instance, cytokines and ROS and RNS [4]. Thus, in the last years, a growing number of studies are being developed to identify effective agents capable of preventing and treating infectious processes in the skin [5].

Similarly, acute and chronic joint diseases, for instance, arthritis and arthrosis, respectively, are modulated by inflammatory processes [6]. These inflammatory processes are also modulated by cytokine synthesis and release, activating degradative enzymes, such as different matrix metalloproteinases (MMPs), which present a central role in the physiopathology of arthritis and arthrosis [7]. Furthermore, this inflammatory cascade is centrally regulated by increased amounts of nitric oxide (NO), concomitantly with epigenetic regulation [7]. From the cellular point of view, chondrocytes can release massive amounts of cytokines, presenting a key role in the physiopathology of arthritis [8]. In the same direction, macrophages are also hyperactivated in arthritis, also releasing cytokines, MMPs, and NO, contributing to disease perpetuation [9].

Natural products have played an important role in the prevention and treatment of human diseases for thousands of years, and in recent decades, great efforts have been made to make natural products more effective and less toxic. Among the plant species present in the Brazilian territory, especially in the Amazon rainforest, we can find *Uncaria tomentosa*, known as cat's claw [10], a potential natural agent with anti-inflammatory activity, capable of reducing, for example, the expression of TNF-alpha in monocytes [11]. In addition, the specie *Endopleura uchi*, known as yellow uxi [12], is also found, which presents a marked antioxidant and anti-inflammatory potential [13]. Thus, many other pharmacologically active plant species with anti-inflammatory potential have been also investigated [14].

Miodesin™, a patented phytocomplex prepared by Fagron Pharmaceutical™ (Brazil) from *Uncaria tomentosa*, *Endopleura uchi*, and astaxanthin, a natural antioxidant carotenoid, has been shown to exert anti-inflammatory effects [15]. In 2018 and 2019, Maia et al. published the first manuscripts showing the role of Miodesin™ in reducing pelvic pain in patients with endometriosis and leiomyoma, confirming the central role of inflammation in the pathogenesis of endometriosis. In the same way, these findings support the role of anti-inflammatory herbal medicines in the treatment of this disease [16, 17]. Therefore, the present study is aimed at evaluating the anti-inflammatory activity of Miodesin™, using different cell lines, besides verifying a possible interaction with epigenetic mechanisms.

## 2. Material and Methods

### 2.1. Reagents

Dulbecco's modified Eagle's medium (DMEM), fetal bovine serum (FBS), penicillin-streptomycin, and phosphate-buffered saline (PBS) were obtained from Gibco BRL (Grand Island, CA, USA). 3-(4,5-Dimethylthiazol-2-yl)-2,5-diphenyltetrazolium bromide (MTT) was purchased from Sigma-Aldrich (St. Louis, MO, USA). The Miodesin™ was supplied by Fagron Pharmaceutical™ (Brazil). The purity and quality of the raw materials used, as well as the formulation of Miodesin™ (*Uncaria tomentosa*, *Endopleura uchi*, and astaxanthin), were monitored by the Fagron Pharmaceutical™ quality control department.

### 2.2. Culture and Cytotoxicity Evaluation of Miodesin by MTT Assay

The human skin keratinocyte cell line (HaCaT) and human chondrocyte cell line (CHON-001) were obtained from the American Type Culture Collection (ATCC, USA). The murine macrophage (RAW 264.7) cells were obtained from the Rio de Janeiro Cell Bank (UFRJ, RJ, Brazil). The cells were cultured in Dulbecco's modified Eagle's medium (DMEM high glucose) supplemented with 10% *v*/*v* fetal bovine serum (FBS), 1% L-glutamine, 100 U/mL penicillin, and 100 mg/mL streptomycin and maintained at 37°C in a humidified atmosphere of 5% CO_2_. The cells were trypsinized every 72 h using 0.01% trypsin and 1 mmol ethylenediaminetetraacetic acid (EDTA). For all the experiments, the Miodesin™ was dissolved in the culture medium in appropriate concentrations. The cell viability of the control and Miodesin™ (1–1.000 *μ*g/mL)-treated chondrocyte, human keratinocyte (HaCaT), and macrophage RAW 264.7 cells was measured using a standard MTT assay. Briefly, 5 × 10^4^ viable cells were seeded into clear 96-well flat-bottom plates (Corning) in the RPMI 1640 medium supplemented with 10% fetal bovine serum (FBS) and incubated with different concentrations of the extract for 24 h. Then, 10 *μ*L/well of MTT (5 mg/mL) was added and the cells were incubated for 4 h. Following incubation, 100 *μ*L of 10% sodium dodecyl sulfate (SDS) solution in deionized water was added to each well and left overnight. The absorbance was measured at 595 nm in a benchtop multimode reader (Molecular Device).

### 2.3. Cell Viability after LPS Treatment

Briefly, cells were seeded in 96-well culture plates at a density of 5 × 10^4^ viable cells/well and incubated for 24 h, then exposed to IC_50_ concentration of Miodesin (200 *μ*g/mL), previously determined in Section 2.1 in the presence of LPS (1 *μ*g/mL) for 60 minutes and incubated for another 24 h with Miodesin at 37°C. The MTT solution was added to the final concentration of 0.5 mg/mL and then incubated for 2 h at 37°C followed by the addition of 0.1 mL of dimethyl sulfoxide to dissolve the MTT-formazan. The amount of MTT-formazan was then determined by measuring the absorbance at 595 nm.

### 2.4. Cytokine and Chemokine Analysis in Cell Culture Supernatants

The concentrations of IL-1*β*, IL-6, IL-8, TNF-*α*, MCP-1 (CCL2), MIP-1*α* (CCL3), and RANTES (CCL5) in the cell culture supernatants were analyzed by using enzyme-linked immunosorbent assay (ELISA) kits (R&D Systems, Minneapolis, MN, USA) following the manufacturer's instructions. Cells were pretreated with LPS (1 *μ*g/mL) for 60 minutes with or without Miodesin (200 *μ*g/mL) for 6 h. The cell culture supernatant (100 *μ*L) was collected to determine the levels of cytokines and chemokines, according to the manufacturer's instructions.

### 2.5. NO Analysis

NO levels were determined by measuring the quantity of nitrite in the cell culture supernatant using a Griess reagent. Chondrocytes, keratinocytes and RAW 264.7 (macrophages) cells were pretreated with LPS (1 *μ*g/mL) for 60 minutes with or without Miodesin (200 *μ*g/mL) for 24 h. The cell culture supernatant (100 *μ*L) was mixed with a 100 *μ*L Griess reagent, and the absorbance was measured at 540 nm. The nitrite concentrations were calculated using a standard calibration curve prepared from different concentrations of sodium nitrite.

### 2.6. Reverse Transcription-Quantitative PCR (RT-qPCR)

Total RNA extracted from cell samples was converted to cDNA using a SuperScript® III RT kit (Invitrogen, Carlsbad, CA), according to the manufacturer's protocol. The concentration of RNA was detected using a NanoDrop 2000 (Thermo Fisher Scientific, Inc.). GAPDH and 18S rRNA were used as the internal control. The thermocycling conditions were as follows: 95°C for 10 min followed by 35 cycles of 95°C for 15 sec and 55°C for 40 sec. The 2^-*ΔΔ*Cq^ method was used to quantify the relative gene expression levels of the target genes. Relative standard curves were generated by serial dilutions, and all samples were run in triplicate.  Table 1 indicates the sense and antisense sequences of primers used in qRT-PCR analysis.

### 2.7. Quantification of the 5-mC Content in Genomic DNA

The genomic DNA cytosine methylation levels in cell lines exposed to Miodesin (200 *μ*g/mL) at 24 h were assessed by using an enzyme-linked immunosorbent assay-based commercial kit (MDQ1, Imprint® Methylated DNA Quantification Kit, Sigma-Aldrich). DNA at a concentration of 150 ng was diluted with 30 *μ*L of binding buffers and incubated at 60°C. The samples were incubated with capture and detection antibodies, and the absorbance was read at 450 nm. Quantification of DNA methylation was obtained by calculating the amount of methylated cytosines in the sample relative to methylation in a positive control, which was provided by the manufacturer.

### 2.8. Statistical Analysis

The obtained results were expressed as mean ± standard error of mean (SEM) from at least three independent experiments, unless stated otherwise. Paired data was evaluated by Student's *t*-test. One-way analysis of variance (ANOVA) was used for multiple comparisons, followed by the Bonferroni test for comparison among the groups. A *p* value of <0.05 was considered significant.

## 3. Results

### 3.1. Effects of Miodesin™ on Cell Viability and on LPS-Induced Cell Cytotoxicity

**Figure 1:**
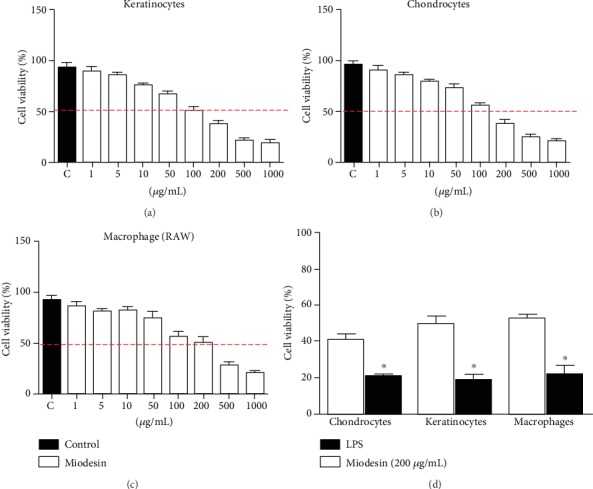
Effects of Miodesin™ on cell line viability and cell toxicity. Cells were treated with different concentrations of Miodesin™ for 24 h, and the IC_50_ was defined as the study test concentration (200 *μ*g/mL) for all cell lines. (a) Chondrocytes, (b) keratinocytes, and (c) macrophages. Effects of Miodesin™ on LPS-induced cytotoxicity. Date shown are representative of three independent experiments. The values are expressed as mean ± SEM, and ^∗^*p* < 0.01 indicates statistical difference (unpaired *t*-test).

Figure 1 shows the different concentrations of Miodesin™, which were tested for cell toxicity to determine the IC_50_ value. The dose of 200 *μ*g/mL was defined as the study dose. Figures 1(a)– 1(c) show the results for keratinocytes, chondrocytes, and macrophages, respectively. In addition, from the IC_50_ dose (200 *μ*g/mL), Miodesin™ was able to reverse the cytotoxicity caused by LPS in all these cell lines (Figure 1(d)).

### 3.2. LPS-Induced Inflammatory Cytokine and Chemokine Release in Cell Lines Is Regulated by Miodesin™

**Figure 2:**
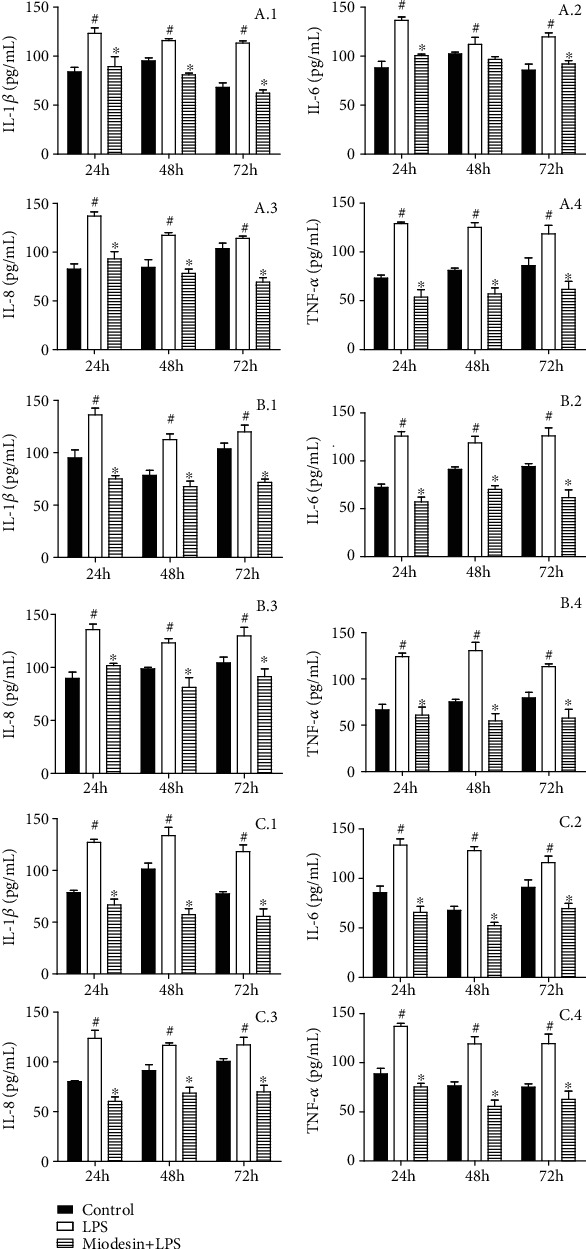
Cells were pretreated with Miodesin™ (200 *μ*g/mL) for 2 h and treated with LPS (1 *μ*g/mL) for 24 h. Values were expressed as mean ± SEM. ^#^*p* < 0.01 vs. control (nontreated cells), and ^∗^*p* < 0.01 vs. LPS-treated cells.

Figure 2 shows the interleukin levels in the supernatant of chondrocytes (Figures 2(A.1) IL-1*β*, 2(A.2) IL-6, 2(A.3) IL-8, and 2(A.4) TNF-*α*), keratinocytes (Figures 2(B.1) IL-1*β*, 2(B.2) IL-6, 2(B.3) IL-8, and 2(B.4) TNF-*α*), and macrophages (Figures 2(C.1) IL-1*β*, 2(C.2) IL-6, 2(C.3) IL-8, and 2(C.4) TNF-*α*). The results demonstrated that while LPS significantly increased the levels of IL-1*β*, IL-6, IL-8, and TNF-*α* for all cell types (^#^*p* < 0.01), Miodesin™ significantly reduced the levels of IL-1*β*, IL-6, IL-8, and TNF-*α* for all cell types (^∗^*p* < 0.01).

**Figure 3:**
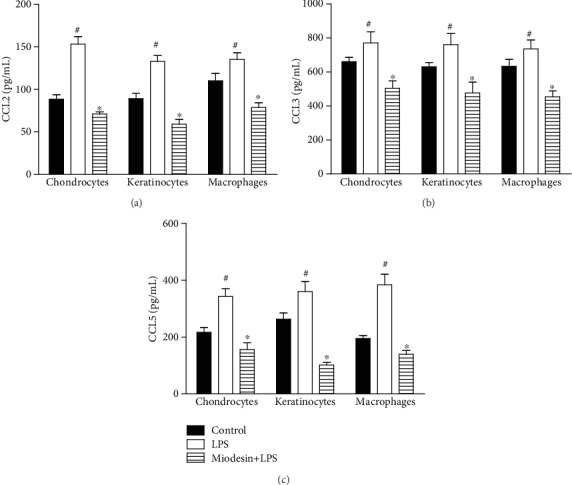
Cells were pretreated with Miodesin™ (200 *μ*g/mL) for 2 h and treated with LPS (1 *μ*g/mL) for 24 h. Values were expressed as mean ± SEM. ^#^*p* < 0.01 vs. control (nontreated cells), and ^∗^*p* < 0.01 vs. LPS-treated cells.

In addition, Figure 3 shows that all cell types responded similarly to LPS stimulation, since LPS increased the levels of CCL2 (Figure 3(a); ^#^*p* < 0.01), CCL3 (Figure 3(b); ^#^*p* < 0.01), and CCL5 (Figure 3(c); ^#^*p* < 0.01), while Miodesin™ significantly reduced the levels of CCL2 (Figure 3(a); ^∗^*p* < 0.01), CCL3 (Figure 3(b); ^∗^*p* < 0.01), and CCL5 (Figure 3(c); ^∗^*p* < 0.01).

### 3.3. Miodesin™ Inhibits LPS-Induced Nitric Oxide (NO) Release

**Figure 4:**
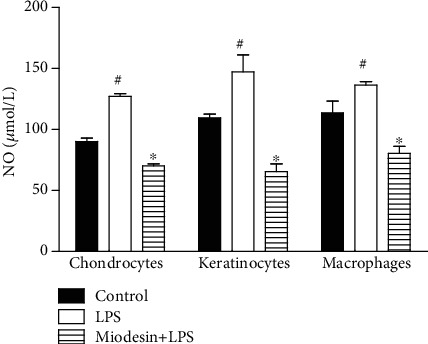
Cells were pretreated with Miodesin™ (200 *μ*g/mL) for 2 h and treated with LPS (1 *μ*g/mL) for 24 h. Values were expressed as mean ± SEM. ^#^*p* < 0.01 vs. control (nontreated cells), and ^∗^*p* < 0.01 vs. LPS-treated cells.

The NO levels released by chondrocytes, keratinocytes, and macrophages are presented in Figure 4. The results demonstrated that LPS significantly induced NO release by chondrocytes (Figure 4, ^#^*p* < 0.01), keratinocytes (Figure 4, ^#^*p* < 0.01), and macrophages (Figure 4, ^#^*p* < 0.01), while Miodesin™ abolished such effects in all cell types tested: chondrocytes (Figure 4, ^∗^*p* < 0.01), keratinocytes (Figure 4, ^∗^*p* < 0.01), and macrophages (Figure 4, ^∗^*p* < 0.01).

### 3.4. Expression of Cytokine and Inflammatory Mediators at mRNA Levels in Cell Lines

**Figure 5:**
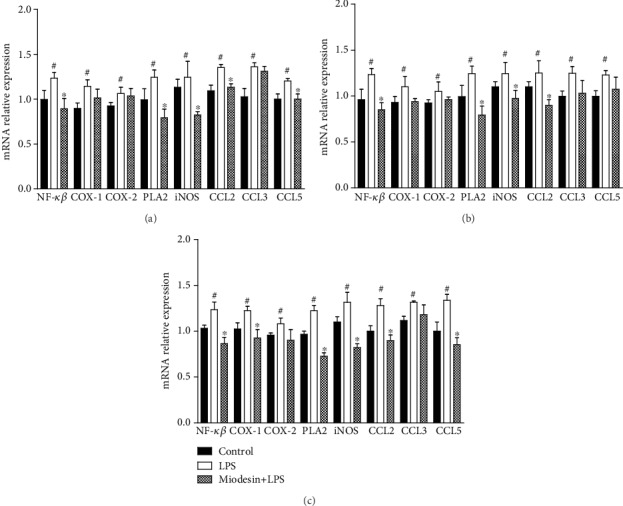
Effects of Miodesin™ on the expression of mRNA of the nuclear transcription factor (NF-*κβ*), inflammatory enzymes (COX-1, COX-2, PLA2, and iNOS), and chemokines (CCL2, CCL3, and CCL5) in chondrocytes (a), keratinocytes (b), and macrophages (c). Values are expressed as mean ± SEM. ^#^*p* < 0.01 vs. control (nontreated cells), and ^∗^*p* < 0.01 vs. LPS-treated cells.

Figure 5 shows the effects of Miodesin™ on the mRNA levels of the nuclear transcription factor (NF-*κβ*), inflammatory enzymes (COX-1, COX-2, PLA2, and iNOS), and chemokines (CCL2, CCL3, and CCL5) on chondrocytes (Figure 5(a)), keratinocytes (Figure 5(b)), and macrophages (Figure 5(c)). The results demonstrated that LPS significantly induced the expression of the nuclear transcription factor (NF-*κβ*; ^#^*p* < 0.01), inflammatory enzymes (COX-1, COX-2, PLA2, and iNOS; ^#^*p* < 0.01), and chemokines (CCL2, CCL3, and CCL5; ^#^*p* < 0.01) on chondrocytes (Figure 5(a)), keratinocytes (Figure 5(b)), and macrophages (Figure 5(c)), while Miodesin™ inhibited such effects in all cell types tested: chondrocytes (Figure 5(a), ^∗^*p* < 0.01), keratinocytes (Figure 5(b), ^∗^*p* < 0.01), and macrophages (Figure 5(c), ^∗^*p* < 0.01).

### 3.5. Miodesin™ Promotes Downregulation in Global DNA Methylation and Decreases Dnmt mRNA Levels

**Figure 6:**
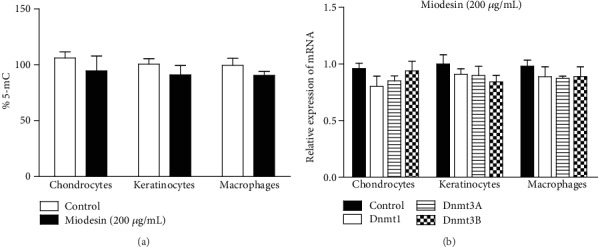
Global DNA methylation and mRNA levels of Dnmts. (a) Methylation status of genomic DNA cytosine methylation in Miodesin™-treated cells. Detection of 5-mC present in the genomic DNA of the control and Miodesin™-treated cells. Methylation was estimated by an enzyme-linked immunosorbent assay-based Imprint® Methylated DNA Quantification Kit (Sigma-Aldrich), which specifically detects 5-mC in the input DNA. Data are represented as percent cytosine methylation as compared with the control. (b) Representative results of fold change difference of Dnmt1, Dnmt3A, and Dnmt3B. Data are expressed as mean ± SEM in three independent experiments, representing the relative levels with normalization by 18S ribosomal RNA. The fold differences were calculated compared with the control groups and shown in the figure. No statistical differences were found.

Cells were treated with 200 *μ*g/mL of Miodesin™; the DNA methylation showed no significant reduction in any of the cell lines evaluated, in comparison to the control at 24 h as estimated using the Imprint® Methylated DNA Quantification Kit (Figure 6(a)). To verify whether the changes observed in DNA methylation was accompanied by changes in the expression of Dnmt genes, the mRNA levels of Dnmt1, Dnmt3A, and Dnmt3B genes in the studied cell lines were quantified after incubation with Miodesin™ 200 *μ*g/mL for 24 h. Miodesin™ did not reduce significantly the endogenous Dnmt1, Dnmt3A, and Dnmt3B mRNA levels in cell lines (Figure 6(b)).

## 4. Discussion

The present study is the first to demonstrate the mechanism of action of Miodesin™ in reducing inflammatory markers such as interleukins, tumor necrosis factor-alpha (TNF-*α*), chemokines, and nitric oxide (NO) that are triggered by the chondrocytes, keratinocytes, and inflammatory cells [18]. In addition, a probable involvement in epigenetic mechanisms may be involved in the anti-inflammatory action of Miodesin™. The presence of the phytocomplex formed by *Uncaria tomentosa* (cat's claw), *Endopleura uchi*, known as uxi, and astaxanthin, the xanthophyll carotenoid, guarantees Miodesin™ anti-inflammatory and antioxidant activity.

The presence of *Uncaria tomentosa* in the formulation gives Miodesin™ a variety of bioactive secondary metabolites, including tetra- and pentacyclic oxindole alkaloids, glycosides, polyoxygenated triterpenes, and procyanidins [19]. Most investigators attribute the biological effects of this plant to the oxindole alkaloids, an assumption that has been corroborated by studies of several such alkaloids that indicated their antioxidant, immunomodulatory, and antineoplastic properties [20]. The anti-inflammatory effects of *Uncaria tomentosa* could be due to its inhibition of lipopolysaccharide- (LPS-) dependent expression of tumor necrosis factor-alpha (TNF-*α*) [11] by reduction of the expression of the transcription factor nuclear factor *κ* light-chain enhancer of activated B cells (NF-*κβ*) [21, 22], an effect that, in turn, regulates the expression of tumor necrosis factor-alpha (TNF-*α*). By reducing the expression of NF-*κβ*, *Uncaria tomentosa* also reduces the TNF-*α* levels and intensifies its anti-inflammatory action [21, 23].

Phytochemical studies with *Endopleura uchi*, which is an Amazon species traditionally used for the treatment of inflammations and female disorders, indicate that the compound bergenin (coumarin) is responsible for the anti-inflammatory action [12, 24], although it indicates the presence of tannins and saponins in the barks [25, 26]. Bergenin, the most abundant molecule, has anti-inflammatory action, apparently through mitogen-activated protein kinase (MAPK) and nuclear factor kappa B (NF-*κβ*) inhibition (N), in addition to inhibiting the action of cycloxygenase-2 [27].

Astaxanthin is a well-known carotenoid, since many studies in recent years have demonstrated its inhibitory role against oxidative stress and inflammation, dangerous processes at the basis of many chronic diseases [28]. Astaxanthin exerts its anti-inflammatory effect by inhibiting nuclear translocation of NF-*κβ* p65 and by preventing ROS accumulation in NRF2-dependent and NRF2-independent mechanisms [29].

The treatment of chondrocytes with Miodesin™ reduced the secretion of interleukins and chemokines, corroborating data found in the literature that show the action of *Uncaria tomentosa* in osteoarthritis [30–33]. It is worth mentioning the reduction in the secretion of two other interleukins, IL-17 and IL-23, that have been found in osteoarthritis joints which also cause destructive proteases and induction of the synthesis of NO [30], in addition to being considered those which play crucial roles in the induction of local inflammation and cartilage destruction diseases such as rheumatoid arthritis [34]. The effects of Miodesin™ on the expression of mRNA of inflammatory enzymes, NF-*κβ*, and chemokines in cell lines in the presence or absence of LPS were also evaluated. NF-*κβ*, PLA2, iNOS, and CCL5 showed significantly reduced mRNA levels, while COX-1, COX-2, and CCL3 showed a reduction, but not significant. It is noteworthy that CCL3 secretion was significantly reduced when chondrocytes were challenged with LPS. The reduction in mRNA expression of the iNOS enzyme, associated with a reduction in NO in the treated chondrocytes, suggests an important role of Miodesin™ in NO modulation [35]. Our results also showed that treatment with Miodesin™ reduced the levels of NF-*κβ* mRNA. Thus, we suggest that Miodesin™ can regulate the function of NF-*κβ*, which has a mechanism associated with the destruction of cartilaginous tissue [36]. Also, in this sense, NF-*κβ* regulates the production of matrix metalloproteinases by chondrocytes that are released promoting matrix degradation [37]. Chemokine CCL5 was increased in chondrocytes after in vitro treatment with LPS. However, when chondrocytes were previously treated with Miodesin™, they showed a significant reduction in the secretion of this chemokine, as well as mRNA levels, suggesting an important role of Miodesin™ in the modulation of this chemokine, which has a primary function of attracting lymphocytes and monocytes as well as other cell types [38, 39] and of activating rheumatoid arthritis synovial fibroblasts (RASFs) to promote MMP-1 and MMP-13 mediated ECM destruction [40].

Keratinocytes form the first line of defense against microorganisms, physical or chemical tissue damage, in addition to producing cytokines that regulate the migration of inflammatory cells, activation of immune responses, and proliferation and differentiation of keratinocytes and fibroblasts [3]. In addition, these cells mediate the skin's immune response by secreting various proinflammatory cytokines [41] and recruit immune cells to the site of insult [42]. Regarding keratinocytes, Miodesin™ reduced the secretion of IL-8, IL-1B, and IFN-*γ*. IL-17 showed a reduction after 72 hours of treatment. Regarding the evaluated chemokines, although all showed a reduction, only CCL2 (MCP-1) showed a significant reduction compared to the control (cells without treatment). CCL2 mRNA levels were also significantly reduced after previous treatment with Miodesin™. Monocyte chemoattractant protein-1 (MCP-1/CCL2) is one of the key chemokines that regulate migration and infiltration of monocytes/macrophages [43]. Upregulated expression of chemokines such as RANTES/CCL5 and MCP-1/CCL2 has been detected in the epidermis of patients with both atopic dermatitis and psoriasis [44]; in addition, with TNF-*α* being a potent inducer of IL-8, whose high production is associated with psoriasis, but not in the skin of patients with atopic dermatitis or healthy skin, we can suggest that, due to the reduced production of TNF-*α* by Miodesin™, the phytocomplex could present itself as an interesting adjunctive therapy option in the treatment of psoriasis [45, 46]. Interestingly, a reduction in IFN-*γ* levels was observed, since IFN-*γ*-activated keratinocytes express a wide range of cytokines, chemokines, and membrane molecules that direct the recruitment, activation, and retention of specific leukocyte subpopulations in the skin [47].

Innate immunity cells, such as macrophages, trigger a rapid immune response, being able to secrete various types of cytokines [48]. In this way, we use RAW 264.7 (macrophages) cells associated with LPS to stimulate these macrophages to produce inflammatory mediators. The results found showed that Miodesin™ inhibited NO production and suppressed iNOS mRNA levels in LPS-stimulated cells. In addition, Miodesin™ also significantly reduced the secretion of cytokines, such as TNF-*α*, IL-6, IL-8, and IL-1*β*. The results of the evaluated chemokines showed that CCL2/MCP-1 CCL3/MIP-1*α* were significantly reduced when compared to RAW 264.7 (macrophage) cells stimulated with LPS. The levels of NF-*κβ*, COX-1, PLA2, CCL2, and CCL5 mRNA were also reduced in RAW 264.7 (macrophage) cells when compared to cells stimulated with LPS. The results obtained suggest that the anti-inflammatory action of Miodesin™ is due, at least in part, to the action of the components of its formulation, either by the action of astaxanthin, reducing proinflammatory cytokine secretion, e.g., IL-1*β*, IL-6, and TNF-*α*, and reducing NF-*κβ* nuclear expression [49, 50]; by the action of *Uncaria tomentosa*, promoting the inhibition of interleukins such as IL-1*β*, IL-17, and TNF-*α*, in addition to the inhibition of NF-*κβ* [10, 51, 52]; or by the action of *Endopleura uchi* [27, 53], inhibiting the action of cyclooxygenases (COX-1 and COX-2) and phospholipase A2 (PLA2), reducing the production of proinflammatory cytokines (IFN-*γ* and TNF-*α*). Our results, therefore, show the anti-inflammatory activity of Miodesin™, by regulating the expression and/or secretion of several important inflammatory biomarkers.

Finally, we evaluated if Miodesin™ promotes changes in the global DNA methylation and in the levels of mRNA of the enzymes called methylases (Dnmt1, Dnmt3A, and Dnmt3B). The results obtained indicated no significant alterations in global DNA methylation in all cell lines evaluated. The levels of mRNA of the enzymes called methylases were not statistically reduced, corroborating the findings of global DNA methylation. These findings seem to be in accordance with the current evidence which suggests a potential role of epigenetics on the level of inflammatory markers reporting on the association of inflammation with global DNA methylation showing a hypomethylation trend [54–56]. The evidence reinforces the role of epigenetic changes in the modification and modulation of transcription factors, leading to the deregulation of multiple cascades of intracellular signaling [57]. In this sense, the literature shows that some bioactive molecules present in components of the formulation of Miodesin™ could have an epigenetic effect, as in the case of hirsutine from the genus *Uncaria*, such as *Uncaria rhynchophylla* [58] and *Uncaria tomentosa* [59], which exerts its epigenetic action inhibiting the activation of NF-*κβ* [58]. Thus, NF-*κβ* could become a possible target in anti-inflammatory therapy, since it is able to regulate epigenetic changes associated with inflammation [60]. The correct understanding of these aspects may generate strategies for the development of new therapeutic approaches, opening space for future studies with the aim of investigating the participation of Miodesin™ in epigenetic mechanisms related to the control of different inflammatory processes.

## Figures and Tables

**Table 1:** 

Gene	Primer sequences
NF-*κβ*	Forward 5′-ATGGCTTCTATGAGGCTGAG-3′
	Reverse 5′-GTTGTTGTTGGTCTGGATGC-3′
COX-1	Forward 5′-AGGAGATGGCTGCTGAGTTGG-3′
	Reverse 5′-AATCTGACTTTCTGAGTTGCC-3′
COX-2	Forward 5′-ACACCTTCAACATTGAAGACC-3′
	Reverse 5′-ATCCCTTCACTAAATGCCCTC-3′
PLA2	Forward 5′-AAAGAACACTATAGGGAGAG-3′
	Reverse 5′-AAAGAGGTAAAGGGCATTGT-3′
iNOS	Forward 5′-CTATCAGGAAGAAATGCAGGAGAT-3′
	Reverse 5′-GAGCACGCTGAGTACCTCATT-3′
CCL2	Forward 5′-GATCCCAATGAGTAGGCTGG-3′
	Reverse 5′-CGGGTCAACTTCACATTCAAAG-3′
CCL3	Forward 5′-ACACCAGAAGGATACAAGCAG-3′
	Reverse 5′-CGATGAATTGGCGTGGAATC-3′
CCL5	Forward 5′-CCCACGTCAAGGAGTATTTCTAC-3′
	Reverse 5′-CTAGGACTAGAGCAAGCGATG-3′
Dnmt1	Forward 5′-GGTTCTTCCTCCTGGAGAATGTC-3′
	Reverse 5′-GGGCCACGCCGTACTG-3′
Dnmt3A	Forward 5′-CAATGACCTCTCCATCGTCAAC-3′
	Reverse 5′-CATGCAGGAGGCGGTAGAA-3′
Dnmt3B	Forward 5′-CCATGAAGGTTGGCGACAA-3′
	Reverse 5′-TGGCATCAATCATCACTGGATT-3′
18S	Forward 5′-AACTGCGGAATGGCTCATTAAATC-3′
	Reverse 5′-TTGATCTGATAAATGCACGCATC-3′
GAPDH	Forward 5′-CGGTGTGAACGGATTTGGC-3′
	Reverse 5′-GTGAGTGGAGTCATACTGGAAC-3′

## Data Availability

The authors declare that all raw data presented in this manuscript will be available upon request.
